# Effectiveness of the Polish program for the treatment of severe allergic asthma with omalizumab: a single-center experience

**DOI:** 10.1186/s12890-016-0224-2

**Published:** 2016-04-26

**Authors:** Izabela Kupryś-Lipińska, Paweł Majak, Joanna Molinska, Piotr Kuna

**Affiliations:** Department of Internal Medicine, Asthma and Allergy, Barlicki University Hospital, Medical University of Lodz, Kopcińskiego 22, 90-153 Łódź, Poland

**Keywords:** Effectiveness, Omalizumab, Severe asthma, Severe asthma exacerbations

## Abstract

**Background:**

A national program for the treatment of severe allergic (IgE-dependent) asthma with omalizumab (OMA) was implemented in Poland in 2013. This observational study evaluated the effectiveness of the Polish OMA program and monitored asthma control after treatment discontinuation.

**Methods:**

In the first year of the program, 53 patients (23 new/30 continuing treatment) received OMA in the Barlicki Hospital, Poland. Patients were evaluated at baseline and after 16 weeks of OMA treatment by spirometry, mean dose of inhaled corticosteroids (ICS) and oral corticosteroids (OCS), number of asthma exacerbations, the Asthma Control Questionnaire (ACQ), and the Asthma Quality of Life Questionnaire (AQLQ). OMA treatment responses were determined using the global effectiveness of treatment evaluation scale. Fourteen patients ceased OMA treatment following ≥36 months of therapy and entered follow up.

**Results:**

All patients treated with OMA *de novo* for at least 16 weeks had a decrease in asthma exacerbations and showed a good (15/16, 94 %) or an excellent (1/16, 6 %) response to treatment. We observed a reduction in OCS dose (≥5 mg/day) in 14/16 (88 %) patients. ACQ and AQLQ scores improved by ≥0.5 points in 15/16 (94 %) and 14/16 (88 %) patients, respectively. After OMA cessation, 11/14 (79 %) patients showed worsening of asthma control and severe exacerbations.

**Conclusions:**

Patients in the OMA program show significant benefits, including reduced use of OCS, improved asthma control and quality of life. After OMA discontinuation, frequent severe exacerbations were observed primarily in patients whose asthma was previously uncontrolled by high OCS doses.

## Background

Omalizumab (OMA) is a biological drug recommended by Global Initiative for Asthma (GINA) experts for the therapy of patients with uncontrolled severe allergic (IgE-dependent) bronchial asthma [1]. Numerous clinical and observational studies have confirmed the efficacy of OMA in improving asthma control, reducing the number and severity of exacerbations, decreasing the need for inhaled (ICS) and oral corticosteroids (OCS) and rescue medications, as well as improving patients’ quality of life (QoL) [2]. In 2003, the American Food and Drug Agency (FDA) recommended OMA for use in patients with moderate-to-severe asthma, and 2 years later the European Medicines Agency (EMA) approved its use in countries of the European Union.

On 17 March 2013, the program for the treatment of severe IgE-dependent bronchial asthma with OMA funded by the National Health Fund (NHF) was implemented in Poland [3]. Previously, OMA was only available to a limited group of patients. Nowadays, the access to the therapy is wider but patients must fulfill strict program qualification criteria (Table 1).

**Table 1:** Comparison of the NHF omalizumab (OMA) treatment program regulations with the drug indications

	OMA program requirements	OMA indications (taken from the European Medicines Agency)
Age	≥12 years	≥6 years
tIgE in serum	30–1500 IU/ml	30–1500 IU/ml
Severe allergic asthma with sensitization to whole-year allergens	Yes	Yes
Uncontrolled asthma despite high-dose ICS plus an additional control drug	ACQ score > 1.5 points (1/6)^a^ >1000 mcg BDP-CFC/day + LABA or LTRA or theophylline	Symptomatic High ICS dose + LABA
Necessity of using OCS continuously or in bursts	Yes minimum of 5 mg of prednisone/day	No
Multiple exacerbations	≥3/year (1/6)^a^	Yes
Hospitalizations due to exacerbations	Yes (1/6)^a^	Not required
Life-threatening asthma attack in medical history	Yes (1/6)^a^	Not required
With airflow limitation	FEV1 < 60 % predicted (1/6)^a^	FEV1 < 80 % predicted
Additional criteria	AQLQ score < 5.0 points (1/6)^a^	Not required
Contraindications	Hypersensitivity to the drug	Hypersensitivity to the drug
Co-morbidities inducing severe course of asthma	Yes^b^	Not contraindicated
Tobacco	Non-smokers – obligatory condition	Not contraindicated
Pregnancy	Absolutely contraindicated	Should not be used during pregnancy unless clearly necessary
Contraindications: simultaneous therapy with immunosuppressive drugs (e.g. methotrexate or cyclosporine), anticancer drugs, immunoglobulin infusions or other biological drugs	Yes	Lack of studies

*Abbreviations*: *ACQ* asthma control questionnaire, *AQLQ* asthma quality of life questionnaire, *BDP-CFC* inhaled beclomethasone CFC, *tIgE* total immunoglobulin E levels, *FEV1* forced expired volume in one second, *ICS* inhaled corticosteroid, *LABA* long-acting beta-adrenoceptor agonist, *LTRA* leukotriene receptor antagonist, *OCS* oral corticosteroid

^a^One of six minor criteria concerning severity and control of asthma; at least three of six criteria have to be fulfilled in order to qualify the patient for the program

^b^Except treated severe allergic rhinitis

The aims of the study were to determine the clinical effectiveness of the OMA treatment program after 16 weeks of therapy in patients receiving the drug for the first time, and to evaluate asthma control after discontinuation of OMA in patients who did not obtain consent for participation in the program or had completed the therapy after 36 months.

## Methods

This is an observational, prospective, single-center study. The patients were diagnosed and treated according to the routine clinical practice and program requirements contained in the Appendix to the Minister of Health Declaration [3].

The Barlicki University Hospital is one of 39 centers across Poland to implement the program. Currently, this hospital has the highest number of patients within the NHF OMA treatment program (i.e., 50 patients or ~19 % of the total number), making it a perfect site for the study. On 4 April 2013, the first patient referred to the program in the Barlicki University Hospital was given the consent of the Qualification Committee for OMA treatment. From 4 April 2013 to 6 April 2014, 53 patients (23 new and 30 continuing treatment, including one who moved from another center) were treated within the OMA program in this hospital. Eleven patients were treated OMA for more than 36 months (i.e., the maximum period of treatment determined by the Qualification Committee [4]) and therefore, were refused enrolment. Three other patients were continuing previous OMA therapy in the program, but were discontinued when they reached 36 months of treatment.

### Evaluation of patients

Patients were evaluated at baseline and after 16 weeks of the treatment in compliance with program requirements contained in the Appendix to the Minister of Health Declaration [3]. On entering the program, a detailed history was collected from each patient, concerning sensitizations, asthma control, exacerbations, pharmacotherapy, complications induced by systemic steroid therapy, and smoking. Each patient underwent resting spirometry, skin prick tests (SPT) when possible, and laboratory tests (including total serum IgE levels, and optionally to SPT, allergen-specific IgE (sIgE) tests), All patients completed the Asthma Control Questionnaire (ACQ) and the Asthma Quality of Life Questionnaire (AQLQ). Moreover the mean daily dose of OCS over the last 6 months, the number of hospitalizations, and the number of severe exacerbations in the previous year were calculated. Monthly exacerbation rate was calculated according following formula: number of exacerbations/months of observations. At the 16^th^ week, early effectiveness was assessed based on the improvement in ACQ and AQLQ scores, severe exacerbations rate, and mean daily dose of OCS. Finally, doctors in charge used a five-point global effectiveness of treatment evaluation (GETE) scale to assess OMA treatment response in our patients, as outlined previously [5]. The early effectiveness was evaluated only in patients who *de novo* received omalizumab to avoid the influence of the previous treatment on the results.

### Inclusion criteria in the OMA program

For inclusion in the OMA program, patients must meet all major qualifying criteria, and a minimum of three minor criteria (from a total of six) of severe uncontrolled allergic bronchial asthma as defined by the NHF (Table 1). The major criteria qualifying patients for the OMA program include: ICS > 1000 mcg/day plus another control drug, and a minimum 5 mg/day of OCS (calculated as a mean dose over the 6 previous months). The minor criteria include: ≥ 3 severe exacerbations in the previous year, hospitalization due to exacerbations in the previous year, a history of life-threatening exacerbations of asthma, ACQ score > 1.5 points, AQLQ score < 5 points, and forced expiratory volume in one second (FEV1) < 60 % predicted.

### Ethics

The study was conducted in accordance with the Declaration of Helsinki [6], and local regulations relating to observational studies. Patients entering the NHF program gave written consent to participate in the program under specific terms for obtaining the medical history, performing the physical examination or additional tests (i.e., lung tests, blood tests, ACQ, AQLQ), and data collection. These terms are described in the Appendix to the Minister of Health Declaration [3]. In addition, patients gave written consent prior to each time the drug was administered, according to the formula developed by the Coordinating Board at the NHF. The study is based on the analysis of data collected prospectively in the program. The source data were encrypted and the extracted data were anonymous. The authors obtained the consent for the study from the ethics committee at the Medical University of Lodz.

### Statistical analysis

Statistical analysis was carried out using the methods of descriptive statistics. The effectiveness of the intervention was analyzed using a one-sample t test and one-sample Wilcoxon test for parametric and nonparametric variables, respectively. Changes in study endpoints were compared with theoretical values that were predefined as clinically important. A *P* <0.05 was considered statistically significant. Analysis was performed on the available data only, according to per protocol approach. There were no missing data for included patients. Analysis was performed using Statistica 8.0 (StatSoft, Inc., Tulsa, USA).

## Results

### Demographic and clinical profiles of patients enrolled in the program

In total, 20 men and 33 women, with a mean age of 46 years, were included in the study. The detailed demographic and clinical data are presented in Table 2. Briefly, the average duration of severe asthma in patients enrolled for the program was 13 years. Allergies were confirmed by the SPT or sIgE tests, and revealed that the house dust mite was the most frequently sensitizing allergen (50/53 patients, 94 %) followed by cat allergen (36/53 patients, 68 %). The mean ICS dose converted to beclomethasone CFC (BDP-CFC) equivalent was 2900 mcg/day, and the mean OCS dose in prednisone equivalent was 16 mg/day (note: the mean was calculated from data over the previous 6 months). In the year preceding enrolment, the mean number of exacerbations in the group was 5.4/year, and 24 patients (45 %) were hospitalized due to exacerbations.

**Table 2:** Demographic and clinical characteristics of patients who qualified for the NHF omalizumab (OMA) treatment program

Patients in the program	All (*n* = 53)
Age, years	
Mean ± SD	45.8 ± 15.3
Min/Max	16/78
Men, n(%)	20(38 %)
Occupational status, n (%)	
Occupational activity	23 (43 %)
Sickness pension	7 (13 %)
Duration of severe asthma, years	
Mean ± SD	13 ± 10.8
Median	10
Min/Max	1/52
Allergy, n (%)	
Dust mites	50 (94 %)
Moulds	19 (36 %)
Cat	36 (68 %)
Dog	23 (43 %)
Other animals	12 (23 %)
Serum tIgE, IU/ml	
Mean ± SD	251 ± 219
Median	174
Min/Max	30/922
N ≥ 76 IU/ml, n (%)	40 (76 %)
ICS dose, mcg/day	
Mean ± SD	2912 ± 1262
Median	2500
Min/Max	1500/8500
OCS dose, mg/day	
Regularly used^a^, n (%)	38 (72 %)
Mean	16 ± 12.6
Median	10
Min/Max	0/50
Complications after OCS, n (%)	30 (57 %)
Arterial hypertension	10 (19 %)
Diabetes	8 (15 %)
Osteoporosis	7 (13 %)
Cataract	3 (6 %)
Glaucoma	2 (4 %)
Cushingoid appearance	9 (17 %)
Adrenocortical insufficiency	1 (2 %)
Other	4 (8 %)
Additional control drugs, n (%)	
LABA	47 (89 %)
LTRA	39 (74 %)
SAMA/LAMA	16 (30 %)
Theophylline	12 (23 %)
Severe exacerbations/year, n	
Min 3, n (%)	53 (100 %)
Mean ± SD	5.6 ± 4
Median	4
Min/Max	3/24
Hospitalizations in the preceding year, n (%)	24 (45 %)
Life-threatening asthma, n (%)	23 (43 %)
ACQ points	
Mean ± SD	3.3 ± 1.1
Median	3.4
Min/Max	1.6/5.4
AQLQ points	
Mean ± SD	3.4 ± 1.2
Median	3
Min/Max	1.6/6.5
FEV1% within normal limits	
Mean ± SD	64.6 ± 26.1
Median	59
Min/Max	23/136

*Abbreviations*: *ACQ* asthma control questionnaire, *AQLQ* asthma quality of life questionnaire, *BDP-CFC* inhaled beclomethasone CFC, *tIgE* total immunoglobulin E levels, *FEV1* forced expired volume in one second, *ICS* inhaled corticosteroid, *LABA* long-acting beta-adrenoceptor agonist, *LAMA* long-acting muscarinic antagonist, *OCS* oral corticosteroid, *SAMA* short-acting muscarinic antagonist, *SD* standard deviation

^a^Continuous intake of at least 6 months

**Fig. 1:**
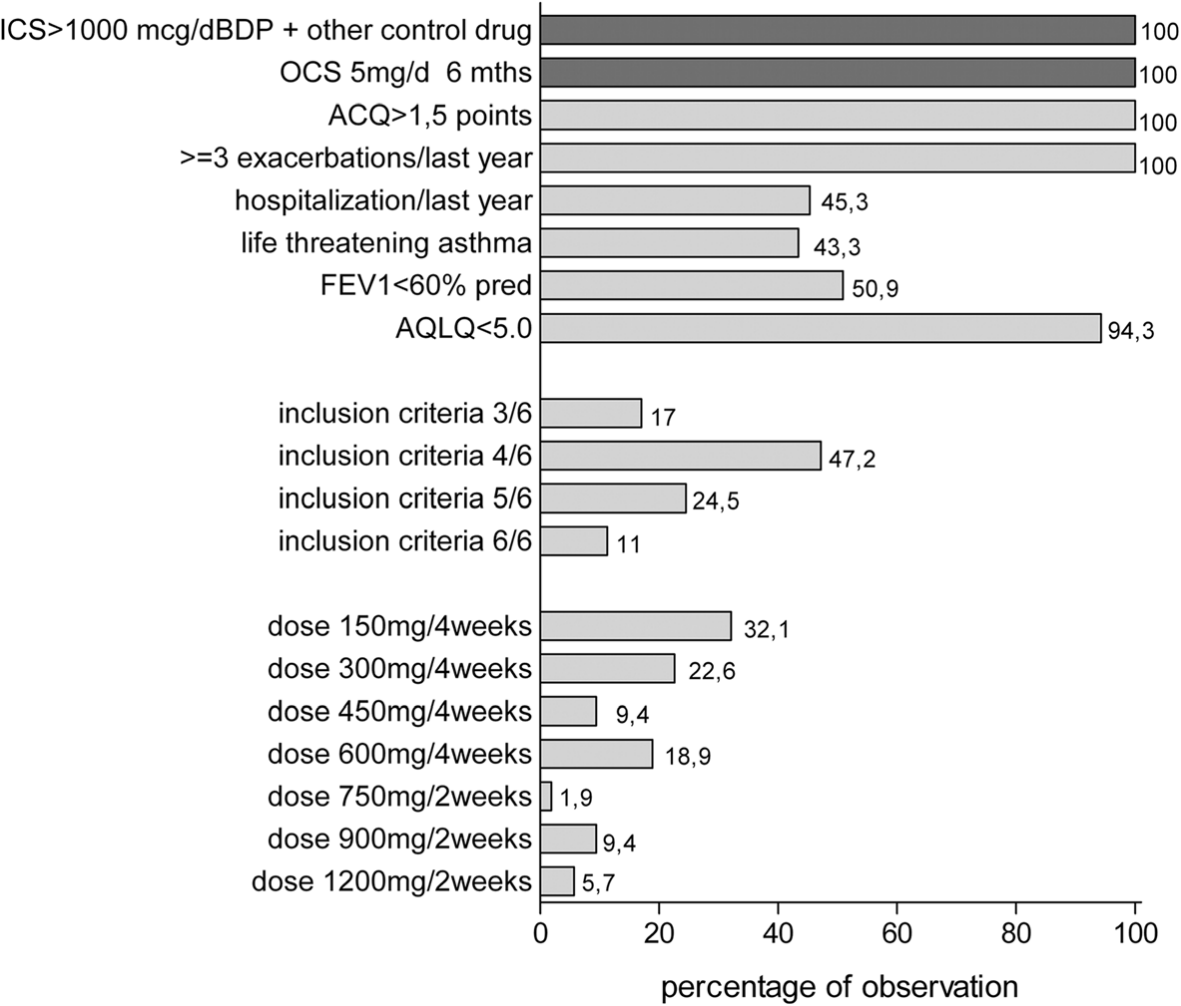
Percentage of study participants who met major and minor criteria for inclusion in the NHF omalizumab (OMA) treatment program. The OMA doses, determined according to the manufacture’s recommendations and the drug characteristics, are shown. (ICS, inhaled corticosteroid; BDP, beclomethasone; SCS, systemic corticosteroids; ACQ, asthma control questionnaire; FEV1, forced expiratory volume in one second)

All patients showed high mean daily dose of OCS at the baseline (Table 2, Fig. 1). Patients most frequently met 4/6 (47.2 %) of the minor criteria (Fig. 1). The most frequently fulfilled minor criteria were: ACQ score > 1.5 points, exacerbations ≥ 3 in the previous year, and AQLQ score < 5.0 points (Fig. 1). The percentage of patients who met each particular criterion of severe uncontrolled allergic bronchial asthma is shown in Fig. 1. The OMA doses for patients participating in the program were determined according to the manufacturer’s recommendations and the drug characteristics (Fig. 1).

### Early effectiveness of OMA therapy

**Fig. 2:**
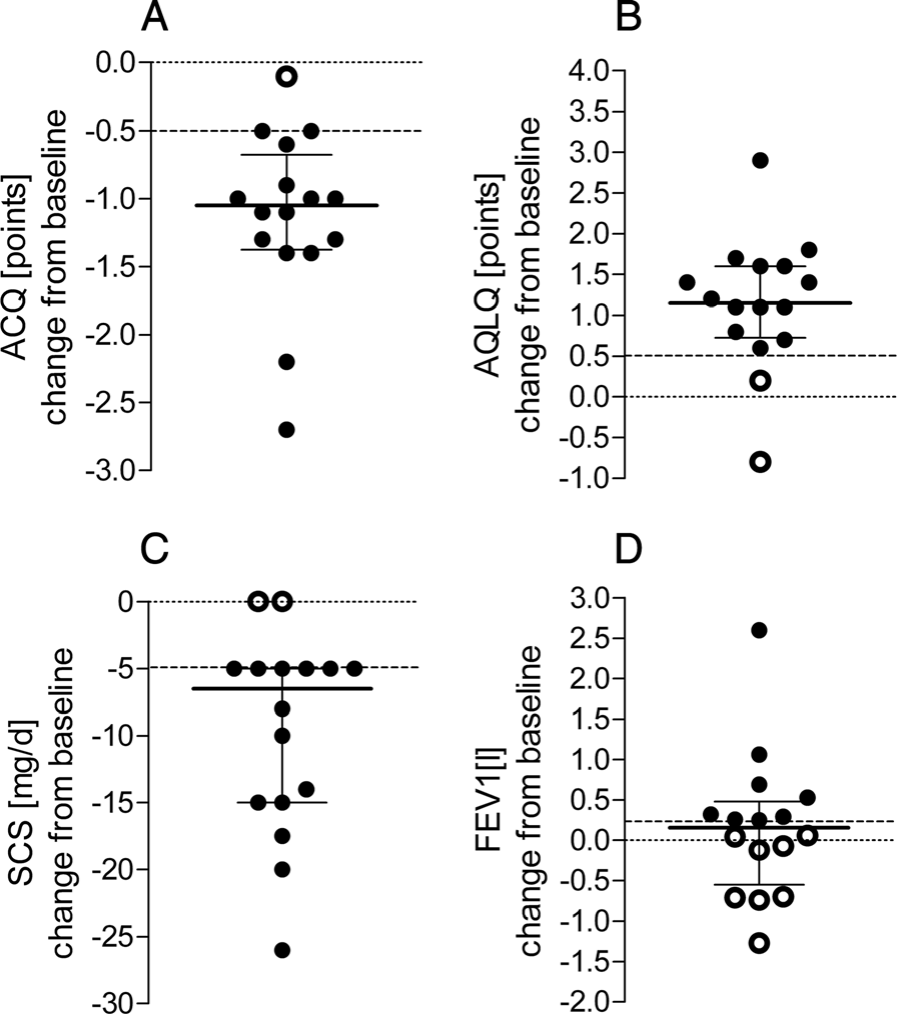
Changes in asthma control questionnaire (ACQ) scores, asthma quality of life questionnaire (AQLQ) scores, systemic corticosteroid (SCS) use, and forced expiratory volume in one second (FEV1) after 16 weeks of omalizumab (OMA) treatment in 16 evaluated patients in the cohort. Dashed lines indicate the cut off for a clinically significance difference

Early effectiveness analysis was conducted in *de novo* patients only. After 16 weeks, 16/23 (70 %) new patients treated with OMA were evaluated. One patient failed to attend the two subsequent meetings and we have lost contact with her, while the other six patients only received OMA treatment for a period shorter than 16 weeks. The evaluation of the effectiveness of the 16-week therapy was carried out in compliance with the program requirements. All patients showed good (15/16, 94 %) or excellent (1/16, 6 %) response to the therapy based on the GETE scale (Table 3). In addition, all patients (100 %) had a decreased number of exacerbations (Table 3). These two criteria are obligatory to warrant ongoing OMA treatment. When evaluating the additional clinical criteria for clinically significant improvement, we found 14/16 (88 %) patients showed a significant reduction (*P* = 0.0001) in OCS use by at least 5 mg (Fig. 2, Table 3). In 15/16 (94 %) patients, the ACQ score was significantly reduced (*P* = 0.0012) by at least 0.5 points, and in 14/16 (88 %) patients, the AQLQ score was significantly improved (*P* = 0.0055) by at least 0.5 points (Fig. 2, Table 3). Of these additional criteria, 100 % of patients met at least two out of the three criteria, and all criteria (3/3) were met by 13/16 patients (81 %). Finally, 8/16 (50 %) patients achieved a clinical improvement in lung function, i.e., showed a ≥ 200 ml improvement in the FEV1, although for whole group the increase in FEV1 was not statistically significant (*P* = 0.5521) (Fig. 2, Table 3).

**Table 3:** Preliminary effectiveness of omalizumab treatment in patients within the NHF program

	Baseline (*n* = 16)	After 16 weeks (*n* = 16)	Mean differences (95 % CI)	Responders^b^, n(%)	P level^c^
Monthly exacerbation rate^a^					
Mean ± SD	0.36 ± 0.14	0.06 ± 0.11	0,30 (0.20 to 0.40)	16 (100 %)	<0.0001
Median	0.33	0			
Min/Max	0.25/0.67	0/0.25			
OCS, mg/day					
Mean ± SD	12.2 ± 7.5	2.5 ± 5.1	−9,72 (-8.68 to -0.75)	14 (88 %)	0.0001
Median	10	0			
Min/Max	5/25	0/20			
ACQ, points					
Mean ± SD	3.54 ± 0.99	2.41 ± 1.18	−1,13 (-0.97 to -0.29)	15 (94 %)	0.0012
Median	3.7	2.65			
Min/Max	1.9/5	0.7/4.1			
AQLQ, points					
Mean ± SD	3.00 ± 0.71	4.15 ± 1.11	1,15 (0.22 to 1.08)	14 (88 %)	0.0055
Median	2.95	3.75			
Min/Max	1.8/4.1	2.9/6.7			
FEV1% predicted					
Mean ± SD	67.6 ± 23.8	73.4 ± 18.9	6,69 (13.27 to 11.64)	8 (50 %)	0.5521
Median	68	76			
Min/Max	31/107	41/104			
GETE: Excellent/Good, n%)				1 (6 %)/15 (94 %)	

^a^ calculated as number of exacerbation/months of observations

^b^ responders were defined as patients with reduced number of exacerbation and or improvement above predefined differences in study end-points: -5 mg, -0.5 points, 0.5 points, 0, 2 l for OCS, ACQ, AQLQ, and FEV1, respectively

^c^comparisons with predefined (clinically important) differences

*Abbreviations*: *ACQ* asthma control questionnaire, *AQLQ* asthma quality of life questionnaire, *FEV1* forced expired volume in one second, *GETE* global effectiveness of treatment evaluation scale, *OCS* oral corticosteroid, *SD* standard deviation

### Evaluation of asthma control after OMA discontinuation

At the time of the program implementation in the Barlicki University Hospital, 31 patients with asthma were receiving OMA treatment. While applications for therapy continuation were submitted for all patients, 11 patients were disqualified as their OMA duration had exceeded 36 months. However, in 9/11 (82 %) patients, OMA treatment was reintroduced due to worsening of asthma control. One patient did not develop severe exacerbation, despite worsening of asthma control. Another patient, with severe exacerbation, did not arrive for the final visit of the qualification procedure. A detailed analysis of this group of patients was presented previously [7].

Twelve patients were admitted to the program temporarily (until they reached a total of 36 months of treatment). Three of these 12 patients have now finished OMA treatment. In 2/3 (67 %) patients who finished the treatment, worsening of asthma control and severe exacerbations were recorded upon OMA cessation. These patients went through the qualification procedure again; one has been given consent for further treatment and another one is waiting for consent. The third patient has failed to communicate with us.

Overall 11/14 (79 %) patients whose OMA therapy was interrupted after ≥ 36 months of treatment showed worsening of asthma control and severe exacerbations.

## Discussion

Patients in the Polish NHF program show significant benefits (good clinical effectiveness), including reduced use of OCS and severe exacerbation rate, improved asthma control and quality of life. Unfortunately after OMA discontinuation, frequent severe exacerbations were observed primarily in patients whose asthma was previously uncontrolled by high OCS doses.

This is the first early report presenting data on the effectiveness of OMA treatment within the Polish NHF program. The data were collected from one site only, but including 20 % of all patients participating in the program (the largest site in Poland). Besides, all of these patients had to fulfilled the same inclusion criteria, thus the population was uniform and the data should be representative for Poland. It must be stressed that only a limited number of patients qualified for the program due to the high price of the drug and the necessity to optimize costs in relation to clinical outcomes. All patients in our study adhered to strict NHF program qualification criteria. These qualification criteria differ from that of the OMA drug indications (Table 1) and the inclusion criteria applied for observational studies [8–21]. Therefore, when comparing the results of this study to those of others, it is important to note these differences. In particular, the age of the patients, the parameters describing severe uncontrolled asthma, and some exclusion criteria are different for NHF treatment program qualification compared to traditional OMA drug indications.

First, the minimum age limit for qualification in the NHF program (12 years) differs from the OMA drug characteristics (6 years) (Table 1). While clinical studies have been performed in a patients as young as six, they were not included in the NHF program due to uncertainty about the pharmaco-economic factors in this age group. Second, all our patients in the NHF program used OCS chronically or frequently. This means that only severely ill patients, who had not responded to OCS, were included. Due to this prolonged OCS use, a high percentage (57 %) of patients presented with diseases relating to OCS effects, including difficult to control hypertension, Cushingoid, diabetes, and osteoporosis (Table 2). The remaining criteria for inclusion in the program were taken from major and minor American Thoracic Society (ATS) criteria for detection of refractory asthma [22]. These criteria are not covered in the drug characteristics, apart from high doses of ICS in combination with another drug to control asthma and impairment of lung ventilation. However, the degree of ventilation impairment in the NHF program criteria is more severe than in the drug characteristics or ATS criteria.

As the NHF program qualification criteria for OMA treatment are more restrictive compared to the clinical and observational studies carried out in other countries, our study cohort is characterized by a more severe course of asthma [8–21, 23]. At baseline, our study group is most similar to the French cohort study [8], which began before OMA was registered in the European Union. In the French study, OMA therapy was administered to patients suffering severe and chronic asthma who did not respond to standard treatment. Our study cohort also shows a similar asthma severity at baseline to the British cohort study [19], for which patients were qualified according to previous criteria endorsed by the National Institute of Health and Care Excellence (NICE) and the National Health Service (NHS).

By comparing clinical data (ACQ, AQLQ, OCS dose, frequency of exacerbations, and hospitalizations) at baseline to that gathered after 16 weeks of OMA therapy, we showed that the implementation of the OMA treatment program in Poland has high clinical effectiveness in patients with severe bronchial asthma. This is similar to other studies, which have shown that OMA therapy results in an improvement in asthma control, QoL, requirement for systemic corticosteroids, and a decrease in frequency of severe exacerbations [9, 14, 17, 18]. However, it is difficult to directly compare our results to those obtained in other studies due to differences in the evaluation of clinical parameters.

The clinical effect of OMA begins from 12–16 weeks and is maintained in over 90 % of treated patients (91.4 %) over subsequent weeks [24]. Similar conclusions were drawn in other real life studies [14, 25]. Therefore, evaluating OMA efficacy at 16 weeks is justified. In our study, we used the GETE scale to evaluate the OMA treatment response at 16 weeks. In the doctor’s opinion, all patients in the Polish OMA treatment program showed either excellent or good treatment response. This is in agreement with the results from across Poland; during first year of program OMA treatment was discontinued in only 9 of 278 qualified patients due to the lack of adequate response to the treatment or side effects [26]. However, it is important to note that the percentage of patients who were evaluated after 16 weeks of treatment was not noted [26].

Compared to our study that showed 100 % OMA response, the percentage of responders (in the GETE scale) in the INNOVATE study was only 61 % [27]. Similarly, when data from seven clinical studies (published between 2001 and 2005) was analyzed, ~60 % OMA treatment efficacy was observed [28]. On the other hand, results of other real life studies revealed a higher level (70–84 %) of OMA treatment efficacy [9, 14, 17, 18]. These differences in OMA efficacy may be due to the fact that OMA therapy provides the best advantage for more severe asthma patients [29]. Such patients may have been excluded from some previous clinical studies. However, as indicated earlier, patients within the Polish NHF OMA treatment program were characterized by more severe asthma than in other cohorts, and therefore, the high clinical effectiveness of OMA therapy (100 % response rate) in our cohort is not surprising.

The optimal duration for OMA therapy remains unclear, although it has been estimated theoretically to be 5 years [30]. In the Polish program, OMA therapy is anticipated for 2 years but is not administered longer than 3 years. To date, only a few studies have evaluated asthma control after OMA discontinuation. We previously reported that following OMA cessation, some patients show a gradual worsening of asthma control and an increase in severe asthma exacerbations after only a short period (i.e., 7.56 ± 2.67 weeks) [7]. Similarly, a relapse of symptoms after OMA cessation was observed in the INNOVATE study [31]. In another study of 61 patients whose OMA treatment was discontinued after almost 2 years (22.7 ± 13.1 months), 55.7 % (34/61) of patients showed worsening of asthma control (mean time to the loss of control was 20.4 ± 2.6 months) [32]. OMA was reintroduced in 59 % (20/34) of these patients, but secondary resistance to OMA was noted in 20 % (4/20) of cases. Although we did not observe a secondary lack of response to OMA treatment in our study, it did take longer for some patients to improve, and the improvement was not as significant as when OMA was used for the first time.

Only one other study by Nopp et al. has evaluated the stability of continuous OMA therapy [33]. They studied patients with severe asthma who were treated with OMA for 6 years, and subsequently observed for a 3-year period. None of these patients had previously used OCS continuously. All the patients responded well to the treatment, and a 6-year stability of the OMA therapeutic effect was observed [33]. While asthma condition worsened in 6/18 (33 %) of patients 3-years after OMA cessation, the majority (12/18, 67 %) of patients reported an improvement in or an unchanged asthma condition [33]. This is different to our study where we observed a gradual worsening of asthma control upon OMA cessation in the majority of patients. However, our cohort of patients differed from other studies with regards to the severity of asthma at the time of enrolment and upon treatment cessation. Therefore, apart from the duration of the therapy, the course of asthma, the level of response to treatment, and other clinical features, can affect the stability of the response to OMA after its cessation. This indicates that decisions regarding cessation of OMA treatment should be undertaken individually, taking into consideration the benefits and risks.

It is estimated that about 1000 severe allergic asthma patients in Poland could potentially be treated with OMA [34], but due to the current strict inclusion criteria many patients may not receive the necessary treatment. In addition, GINA and expert panel report 3 (EPR-3) recommendations suggest that OMA should be included prior to regular OCS treatment. Therefore, in order to bring the Polish OMA treatment program in line with the rest of the European Union, the Group of Experts of the Polish Society of Allergology have proposed that the minimum age limit could be lowered, the requirement for earlier treatment with systemic corticosteroids used continuously or nearly continuously could be abolished, and the FEV1 threshold value could be raised [35]. Such measures would see more patients benefit from the program.

## Conclusions

The implementation of the Polish OMA program has brought the treatment of severe asthma in Poland closer to standards applied in other countries of the European Union. The experiences gained by the physicians working in the Barlicki University Hospital during the first year of the program demonstrate that patients significantly benefit from OMA treatment, which justifies the continuation of the program.

### Availability of data and materials

The dataset supporting the conclusions of this article is presented within the article. The detailed clinical data set is not publically available to protect research subject privacy and confidentiality.

## Abbreviations

ACQ: asthma control questionnaire
AQLQ: asthma quality of life questionnaire
ATS: American Thoracic Society
BDP-CFC: dose converted to beclomethasone CFC
EMA: European Medicines Agency
FDA: Food and Drug Agency
FEV1: forced expiratory volume in one second
GETE: global effectiveness of treatment evaluation
GINA: global initiative for asthma
ICS: inhaled corticosteroids
NHF: National Health Fund
NHS: National Health Service
NICE: National Institute of Health and Care Excellence
OCS: oral corticosteroids
OMA: Omalizumab
QoL: quality of life
SCS: systemic corticosteroid
sIgE: allergen-specific IgE
SPT: skin prick tests
